# Quorum Sensing and Metabolic State of the Host Control Lysogeny-Lysis Switch of Bacteriophage T1

**DOI:** 10.1128/mBio.01884-19

**Published:** 2019-09-10

**Authors:** Leanid Laganenka, Timur Sander, Alexander Lagonenko, Yu Chen, Hannes Link, Victor Sourjik

**Affiliations:** a Max Planck Institute for Terrestrial Microbiology and LOEWE Center for Synthetic Microbiology (SYNMIKRO), Marburg, Germany; b Faculty of Biology, Belarusian State University, Minsk, Belarus; University of Minnesota Medical School

**Keywords:** *Escherichia coli*, bacteriophage lysis, cyclic AMP, quorum sensing

## Abstract

Bacterial viruses, or bacteriophages, are highly abundant in the biosphere and have a major impact on microbial populations. Many examples of phage interactions with their hosts, including establishment of dormant lysogenic and active lytic states, have been characterized at the level of the individual cell. However, much less is known about the dependence of these interactions on host metabolism and signal exchange within bacterial communities. In this report, we describe a lysogenic state of the enterobacterial phage T1, previously known as a classical lytic phage, and characterize the underlying regulatory circuitry. We show that the transition from lysogeny to lysis depends on bacterial population density, perceived via interspecies autoinducer 2. Lysis is further controlled by the metabolic state of the cell, mediated by the cyclic-3′,5′-AMP (cAMP) receptor protein (CRP) of the host. We hypothesize that such combinations of cell density and metabolic sensing may be common in phage-host interactions.

## Abstract (precis)

The dynamics of microbial communities are heavily shaped by bacterium-bacteriophage interactions. But despite the apparent importance of bacteriophages, our understanding of the mechanisms controlling phage dynamics in bacterial populations, and particularly of the differences between the decisions that are made in the dormant lysogenic and active lytic states, remains limited. In this report, we show that enterobacterial phage T1, previously described as a lytic phage, is able to undergo lysogeny. We further demonstrate that the lysogeny-to-lysis decision occurs in response to changes in the density of the bacterial population, mediated by interspecies quorum-sensing signal AI-2, and in the metabolic state of the cell, mediated by cAMP receptor protein. We hypothesize that this strategy enables the phage to maximize its chances of self-amplification and spreading in bacterial population upon induction of the lytic cycle and that it might be common in phage-host interactions.

## INTRODUCTION

Dynamics of environmental microbial communities are shaped by the ability of bacteria to promptly detect and respond to a variety of abiotic and biotic factors on both the individual and group levels. These include sensing of nutrient availability and internal metabolic state by individual cells, mediated by cyclic-3′,5′-AMP (cAMP), a universal second messenger broadly used by both prokaryotes and eukaryotes (1, 2). In Escherichia coli and other bacteria, cAMP signaling is mediated by a global transcription regulator, cAMP receptor protein (CRP), which can act both as an activator and a repressor of gene expression in its cAMP-bound form (1). CRP is known to regulate allocation of cellular resources to biosynthesis, utilization of alternative carbon sources, motility, stress response, biofilm formation, and pathogenicity (1, 3–8). Cellular levels of cAMP are controlled by the influx of glucose or other carbon sources into the cell, at least partly coupled to their phosphorylation-dependent uptake through the phosphotransferase system (PTS) (7, 9).

Bacteria are further capable of regulating their collective behaviors in response to changes in population density and species composition (10, 11), by secreting and responding to small-molecule autoinducers (AIs). These quorum-sensing molecules enable communication both within and between species, with the interspecies signaling being primarily mediated by autoinducer 2 (AI-2) (12).

Given the importance of these sensory systems for bacterial growth and survival, they are likely to be hijacked by bacterial viruses (bacteriophages [phages]; reviewed in reference 13) to control their proliferation, survival, and dissemination. In accordance with the two prototypical strategies of phage proliferation, lytic (virulent) phages always rely on rapid replication and lysis of the infected host cells, whereas lysogenic (temperate) phages can either enter the lytic cycle or a dormant lysogenic cycle within their host cells. While the lysogenic prophage state can be stable for many host generations, the prophage can also become induced to reenter the lytic cycle in a manner dependent on intra- and extracellular conditions. The regulation underlying this decision has been extensively studied for the enterobacterial phage lambda, which integrates into the host chromosome but can excise itself and initiate replication in response to poor nutrition and DNA-damaging stress (14, 15). A number of other lysogenic phages have been described previously also, some of which do not integrate into the host genome but exist extrachromosomally (16–20).

Despite the well-recognized importance of bacteriophages in microbial ecosystems, our understanding of mechanisms controlling phage dynamics in bacterial populations, and particularly our understanding of the lysogeny-lysis decisions, remains limited. While most of the early phage work focused on the mechanisms that enable phages to enter and replicate in individual cells, several recent studies revealed complex behaviors within phage populations, including cooperation during infection (21, 22), as well as communication between phages and perception of the host density (23–25), in making the choice between lysogeny and lysis.

In this report, we describe a novel lysogenic state of E. coli phage T1, previously described as a typical lytic phage. Induction of the lytic state of this prophage depends on both AI-2-based signaling and metabolic sensing mediated by cAMP, through an interplay between the phage-encoded transcription regulator Pir (Orf23) and the host CRP. We hypothesize that such integration of metabolic and quorum-sensing cues may be common in dynamic interactions between populations of phages and their hosts.

## RESULTS

### E. coli ATCC 14155 carries an AI-2- and glucose-inducible prophage.

**FIG 1:**
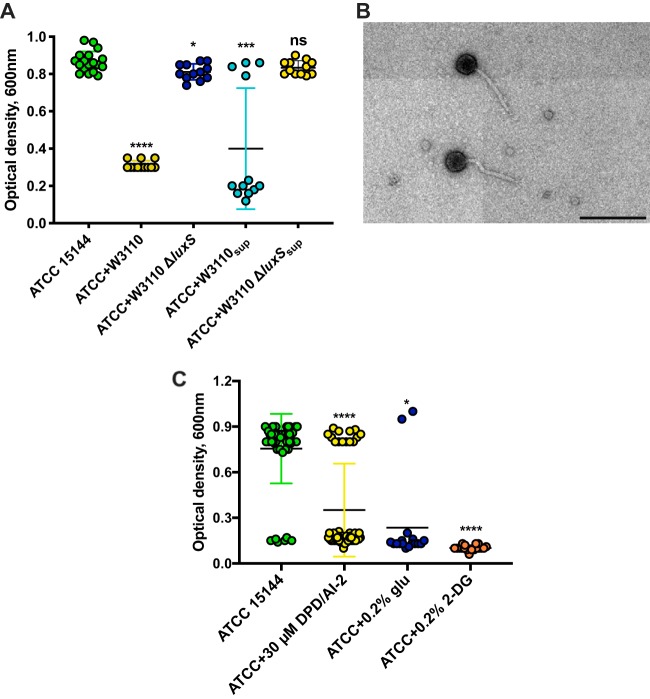
E. coli ATCC 14155 carries an AI-2- and sugar-inducible prophage. (A) Optical densities of E. coli ATCC 14155 cultures (each dot represents individual culture) grown alone or in a 1:1 mixture with E. coli W3110 or W3110 Δ*luxS* bacteria or with 10 μl E. coli W3110 wild-type bacteria or Δ*luxS* cell-free supernatants. (B) Transmission electron microscopy (TEM) of phage particles found in E. coli ATCC 15144 lysates. Scale bar, 200 nm. Note that the different levels of brightness in the four quadrants of the image represent an artifact of the TEM imaging system. (C) Prophage induction in E. coli ATCC 14155 by AI-2 and sugar influx. Single dots represent individual cultures. glu, glucose; 2-DG, 2-deoxy-d-glucose. Means of results of a minimum of 12 independent replicates are shown; error bars represent standard deviations. *P* values were calculated using the Mann-Whitney test (****, *P < *0.0001; ***, *P < *0.0005; *, *P < *0.05; ns, not significant).

In coculturing different E. coli isolates with the laboratory E. coli strain W3110 in liquid tryptone broth (TB), we observed that E. coli ATCC 15144 reproducibly lysed after 2 to 3 h in the coculture, although it grew normally in the monoculture (Fig. 1A). Such lysis could also be induced by addition of cell-free supernatants of W3110 to the growing cultures of E. coli ATCC 15144. Because lysis occurred in most but not all cultures in the latter case, we hypothesized that it might have been caused by prophage induction. Indeed, microscopic analysis of the lysates revealed the presence of virion particles (Fig. 1B).

The observed lysis of ATCC 15144 in the presence of W3110 or its supernatant indicated induction by a W3110-secreted signaling molecule. Since AI-2 is the only established quorum-sensing molecule of E. coli, we tested whether lysis occurs in the presence of E. coli W3110 cells deleted for *luxS*, which encodes AI-2 synthase (26). No lysis was observed in this case or upon addition of W3110 Δ*luxS* culture supernatant (Fig. 1A), strongly suggesting involvement of AI-2 in prophage induction.

To further verify this hypothesis, we assessed whether synthetic (S)-4,5-dihydroxy-2,3-pentadione (DPD) would be able to induce the prophage in a similar manner. Since DPD molecules spontaneously convert to AI-2 in solution (12, 27), we refer to DPD here as DPD/AI-2. In analyzing the growth characteristics of a larger number (*n *=* *50) of independent E. coli ATCC 15144 cultures, we observed a low (∼12%) frequency of spontaneous prophage induction even in the absence of any further treatment (Fig. 1C). This lysis rate was dramatically enhanced (reaching ∼70%) by addition of 30 μM DPD/AI-2, a concentration which is in the range of those observed in E. coli cultures (28, 29). Importantly, the growth of E. coli ATCC 15144 was not altered by DPD/AI-2 addition (Fig. 1C), and no growth in minimal medium with DPD/AI-2 as a sole carbon source could be observed (data not shown) (30). We thus conclude that AI-2 signaling, rather than its effect on growth, leads to prophage induction.

We observed that prophage could also be induced in E. coli ATCC 15144 cultures growing in TB by addition of glucose (Fig. 1C). Notably, 2-deoxy-d-glucose (2-DG), a nonmetabolizable analogue of glucose that is nevertheless imported and phosphorylated by the PTS (31, 32), also induced cell lysis (Fig. 1C), indicating that the observed induction was most likely caused by the PTS signaling rather than by glucose metabolism. Generally, prophage induction and cell lysis in response to AI-2 and glucose occurred between 60 and 130 min after addition of the respective compounds to the growing culture (see Fig. S1A in the supplemental material). No lysis was observed before or after these time points during our experiments. Prophage induction resulted in the release of 8 × 10^9^ to 1.15 × 10^10^ phage particles per ml, with no apparent difference in phage titers between spontaneous or AI-2-containing supernatant-mediated or glucose-mediated prophage inductions (Fig. S1B).

10.1128/mBio.01884-19.1 FIG S1 AI-2- and glucose-dependent prophage induction in E. coli ATCC 15144. (A) Time required for lysis of individual E. coli cultures after stimulation with W3110 cell-free supernatant or with glucose. Single dots represent the time between stimulation and visible lysis of the culture. No lysis was observed before 60 min and after 130 min following stimulation. (B) Number of active phage particles formed after spontaneous or induced prophage activation. E. coli ATCC 15144 lysis was induced by addition of W3110 cell-free supernatant or glucose or both. The titer of the phage was calculated in PFU per milliliter using the Gratia method. No PFUs were detected in nonlysed cultures. *P* values were calculated using the Mann-Whitney test (ns, not significant). (C) Extracellular AI-2 in individual or mixed E. coli ATCC 15144 and W3110 cultures and in individual ATCC 15144 cultures upon addition of 5 μM DPD/AI-2 after 1 h of incubation. Extracellular AI-2 was assessed by analysis of P*lsr-gfp* activity of the biosensor that was defective in AI-2 production (see Materials and Methods). The dashed line represents P*lsr-gfp* activity in the absence of AI-2. Means of results from a minimum of eight independent replicates are shown; error bars represent standard deviations. *P* values were calculated using the Mann-Whitney test (***, *P < *0.0005; **, *P < *0.005; *, *P < *0.05; ns, not significant). (D) Optical densities of E. coli ATCC 14155 cultures (each dot represents individual culture) grown alone and with 5 μM DPD/AI-2. *P* values were calculated using the Mann-Whitney test (*, *P < *0.05). (E) Extracellular AI-2 in E. coli ATCC 15144 culture in TB medium grown from 1:100 (1×) and 1:25 (4×) dilutions of the overnight culture. Extracellular AI-2 was measured after 3 h of growth at 37°C with shaking. Means of results from a minimum of four independent replicates are shown; error bars represent standard deviations. *P* values were calculated using the Mann-Whitney test (ns, not significant). Download FIG S1, EPS file, 0.2 MB. Copyright © 2019 Laganenka et al. 2019 Laganenka et al. This content is distributed under the terms of the Creative Commons Attribution 4.0 International license.

Interestingly, the AI-2-dependent prophage induction mechanism seemed to be sensitive to even a small elevation of the AI-2 concentration. While E. coli ATCC 15144 produces AI-2 (Fig. S1C), its extracellular concentration is apparently not sufficient to induce lysis in most cultures in the mid-exponential phase of growth. However, a modestly higher level of AI-2 produced by the W3110 strain in the ATCC 15144:W3110 mixed cultures or addition of 5 μM DPD/AI-2 to growing ATCC 15144 cultures was already enough to activate the lysogeny-lysis switch and cell lysis (Fig. S1C and D). In contrast, increasing the initial ATCC 15144 inoculum size did not result in significant changes in extracellular AI-2 levels in growing cultures (Fig. S1E), most likely due to the balance of AI-2 production and uptake (12), and thus did not lead to increased phage release.

### Phage identification.

Sequencing the phage DNA revealed that it shares 99.86% identity with the enterobacterial phage T1. This was highly surprising, since T1 phage had been described as a classical example of a virulent phage (33–35). Nevertheless, we could invariably detect T1 phage DNA stably propagating within the host E. coli cells without lysis (Fig. S2A). This lysogeny was apparently established in the absence of integration into the bacterial chromosome, as revealed by genome analysis of E. coli ATCC 14155 by the use of both nucleotide BLAST searches against the GenBank database and the phage sequence-seeking PHASTER tool (see Materials and Methods for details). Consistently, no bacterial genome sequences flanking T1 phage genome were observed. Despite its extrachromosomal residence, the prophage is highly stable and ATCC 15144 could not be cured despite repeated sequential restreaking on lysogeny broth (LB) agar plates.

10.1128/mBio.01884-19.2 FIG S2 Lysogeny of T1 phage. (A) T1 phage stably propagates with its host, E. coli ATCC 15144. Colonies of E. coli ATCC 15144 harboring lysogens of T1 phage were repeatedly restreaked on fresh 1.5% LB agar plates for 28 days. The presence of T1 phage DNA was assessed by PCR using primers specific to *pir* (with an expected product size of 453 bp) after 1, 7, 14, and 28 days. PCR products were separated in a 1.0% agarose gel together with a GeneRuler 1-kb DNA ladder. Purified samples of T1 phage and BL21(DE3) DNA were used as positive (+) and negative (-) controls, respectively. The figure is representative of results from 10 independent replicates. (B) Colonies of T1 lysogens formed during T1 infection of E. coli ECOR-12. A 100-μl volume of ECOR-12 overnight culture was spread on the surface of a 1.5% LB agar plate. A 15-μl volume of T1 phage lysate was spotted onto the agar plate, followed by overnight incubation at 37°C. The figure is representative of all T1 phage host strains found in this study. Scale bar, 1 cm. (C) T1 phage lysogeny is not stable in other host strains. Susceptible E. coli strains were infected with T1 phage as described for panel B, and the presence of T1 phage DNA was assessed in the lysogenic colonies appearing within the plaques (C) and in the overnight cultures grown from such colonies (NC). The presence of T1 phage DNA was assessed with PCR using primers corresponding to *orf47* (encoding a major capsid protein; expected size, 913 bp). PCR products were separated in a 1.0% agarose gel together with a GeneRuler 1-kb DNA ladder. A colony of E. coli ATCC 15144 was used as a positive control. WT, wild-type strain. Bands other than the 913-bp fragment correspond to nonspecific amplification products. (D) T1 phage was lost from ECOR-16 and ECOR-50 lysogens during subcultivation. The lysogens of ECOR-16 and ECOR-50 were obtained, and the presence of T1 phage DNA was assessed with PCR as described for panel C. C, lysogeic colony; NC, overnight culture; DC, day culture grown to an OD_600_ of 0.3. (E) *pir* expression in wild-type (WT) and lysogen (*lys*) colonies. Strains ATCC 15144, ECOR-4, ECOR-13, ECOR-16, ECOR-50, and ATCC 11103 were transformed with a plasmid carrying P*pir-gfp*, and *pir* promoter activity and the percentages of *pir*-expressing cells were measured using flow cytometry. All the strains (excluding ATCC 15144) were then lysogenized with T1 phage as described above, and P*pir-gfp* activity was measured in the lysogen colonies obtained. *P* values were calculated using the Mann-Whitney test (**, *P < *0.005; *, *P < *0.05; ns, not significant). Download FIG S2, JPG file, 2.2 MB. Copyright © 2019 Laganenka et al. 2019 Laganenka et al. This content is distributed under the terms of the Creative Commons Attribution 4.0 International license.

Our analysis of the phage genome identified at least three genes that might be associated with the lysogenic lifestyle. Two of these genes, *orf23* and *orf65*, code for putative transcription regulators. A third gene, *orf30*, encodes a homolog of Cor, which is involved in lysogeny of N15 phage, leading to surface exclusion of several bacteriophages, including T1 (20). Homologs of Cor are also found in several other lysogenic phages (36, 37). Consistent with its expected function, overexpression of Cor resulted in resistance to phage infection in an otherwise T1-susceptible host, E. coli ECOR-4 (Fig. S3).

10.1128/mBio.01884-19.3 FIG S3 The T1 phage genome encodes a functional Cor homologue. E. coli ECOR-4 cells (naturally susceptible to T1 phage infection) were transformed with a plasmid carrying the *cor* gene of T1 phage under the control of IPTG-inducible *trc* promoter. Cultures induced with the indicated levels of IPTG were incubated with or without T1 phage lysate. Means of results from at least three independent replicates are shown; error bars represent standard deviations. Download FIG S3, EPS file, 0.1 MB. Copyright © 2019 Laganenka et al. 2019 Laganenka et al. This content is distributed under the terms of the Creative Commons Attribution 4.0 International license.

Apart from ECOR-4, T1 phage was able to infect several other tested E. coli strains, including ECOR-13, ECOR-16, ECOR-50, and ATCC 11303. In all cases, we observed bacterial colonies appearing in the plaque zones formed by the lytic activity of the phage (Fig. S2B). But although T1 phage DNA was initially detected in these colonies, indicating induction of the lysogenic state, these E. coli strains apparently could not support stable lysogeny as the phage DNA was invariably lost during cultivation in liquid TB medium (Fig. S2C and D).

In contrast, although the genome of E. coli ATCC 15144 is nearly identical to that of the common laboratory strain BL21(DE3), the latter could not be infected by the phage. The reason for this selectivity remains to be elucidated, but ATCC 15144 carries several open reading frames (ORFs) absent in BL21(DE3), including ORFs encoding remnants of prophages, an IS3 family transposase, adhesins, and parts of secretion systems (see Table S2 in the supplemental material).

### Phage-encoded transcription regulator Pir controls AI-2- and PTS-dependent prophage induction.

**FIG 2:**
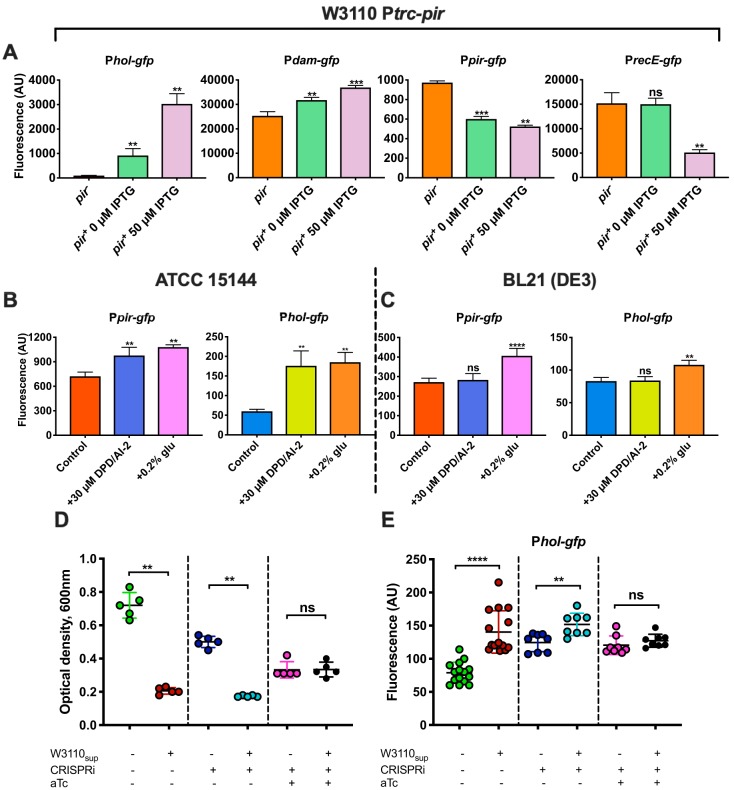
T1 phage-encoded transcription regulator Pir (Orf23) controls AI-2- and sugar-dependent prophage induction. (A) Pir is a functional transcription regulator. Activities of *hol*, *dam*, *pir*, and *recE* promoters controlling *gfp* expression (measured by flow cytometry and expressed in arbitrary units [AU]) in the absence (*pir*^−^) or presence (*pir*^+^) of plasmid-harbored *pir* were measured in E. coli W3110 by flow cytometry. (B) *pir* and *hol* were upregulated during AI-2- and glucose-mediated prophage induction. Activities of *pir* and *hol* promoters were measured at the onset of visible cell lysis by flow cytometry. (C) Activities of *pir* and *hol* promoters in a phage-free background strain, BL21(DE3), were measured by flow cytometry 2 h after addition of 30 μM DPD/AI-2 or 0.2% glucose. (D) Effect of CRISPRi-mediated inhibition of *pir* expression on lysis of E. coli ATCC 15144. Single dots represent optical densities of individual E. coli cultures (CRISPRi^−^ aTc^−^) carrying a CRISPRi system without (CRISPRi^+^ aTc^−^) or with (CRISPRi^+^ aTc^+^) induction of dCas9 protein expression, measured 2 h after addition of 10 μl E. coli W3110 cell-free supernatant. aTc, anhydrotetracycline. (E) *hol* promoter activity measured by flow cytometry in the setup described in the panel D legend. Single dots represent *hol* promoter activities in individual cultures. Means of results from a minimum of three independent replicates are shown; error bars represent standard deviations. *P* values were calculated using the Mann-Whitney test (****, *P < *0.0001; ***, *P < *0.0005; **, *P < *0.005; ns, not significant).

To verify that *orf23* encodes a functional transcription regulator—here renamed Pir (for “prophage induction regulator”)—we expressed Pir from an IPTG (isopropyl-β-d-thiogalactopyranoside)-inducible plasmid in E. coli W3110 strains carrying reporter plasmids containing genes encoding green fluorescent protein (GFP) under the control of several phage promoters. Indeed, we observed that Pir activates promoters of *hol* (*orf13*, coding for holin) and *dam* (*orf20*, coding for Dam methylase) while negatively regulating its own promoter and the promoter of *recE* (*orf29*, coding for putative exodeoxyribonuclease VIII) (Fig. 2A). Since holin is required for cell wall degradation at the end of the lytic cycle (38, 39), the positive regulation of *hol* by Pir indicated an activating role of Pir in the lysogenic-to-lytic transition. Consistent with this role, activities of both the *pir* promoter and the *hol* promoter were upregulated during DPD/AI-2- and PTS-mediated prophage induction in E. coli ATCC 15144 (Fig. 2B). Both promoters were also induced by glucose, but not by AI-2, in the phage-free E. coli BL21(DE3) strain (Fig. 2C; see also Table S2). Changes in *pir* expression were also detected in the majority of T1-susceptible strains upon their lysogenization (Fig. S2E), but while *pir* promoter activity was downregulated in some lysogens, it was upregulated instead or did not change in other lysogenized strains. Thus, *pir* expression does not seem to be directly involved in establishing lysogeny of T1 phage.

To further confirm the function of Pir, we depleted *pir* mRNAs in the host E. coli ATCC 15144 strain with CRISPR interference (CRISPRi). CRISPRi allows sequence-specific inhibition of gene expression by blocking transcription with a deactivated Cas9 (40, 41). In order to deplete *pir* mRNA, cells were cotransformed with two plasmids encoding the anhydrotetracycline (aTc)-inducible dCas9 protein (a mutation of Cas9 without endonuclease activity) and constitutively expressed single guide RNA (sgRNA) targeting the coding region of *pir*. To ensure the effective repression of *pir* transcription, dCas9 expression was induced in both overnight and day cultures of E. coli by addition of 5 ng/ml aTc. The resulting CRISPRi-mediated downregulation of *pir* expression (Fig. S4A) abolished induction of T1 prophage and cell lysis upon addition of AI-2 (in the form of W3110 cell-free supernatant) or glucose (Fig. 2D; see also Fig. S4B and C). Consistently, no upregulation of *hol* expression was observed upon addition of W3110 supernatant (Fig. 2E), although the activity of the *hol* promoter was generally elevated in the presence of the CRISPRi system. This basal elevation was apparently due to the protein burden caused by dCas9 expression, which was also observed in the E. coli W3110 strain carrying no T1 prophage (Fig. S4D and E). Despite its apparent role in the activation of lysis in response to AI-2, the activity of Pir did not seem to be directly affected by AI-2 (Fig. S5).

10.1128/mBio.01884-19.4 FIG S4 CRISPRi-mediated inhibition of *pir* expression. (A) Effective *pir* mRNA depletion by CRISPRi as measured by real-time quantitative PCR (see Materials and Methods). Each quantitation cycle (*C_q_*) value indicates the number of the cycle in which fluorescence can be detected and is therefore inversely proportional to the initial copy numbers of the target. “ND” indicates *C_q_* values that are higher than 30 and which represent results of nonspecific amplification. *cor* mRNA was used as an internal control. Means of results from four independent replicates (two technical replicates each) are shown; error bars represent standard deviations. *P* values were calculated using the Mann-Whitney test (ns, not significant). (B) Single dots represent optical densities of individual E. coli ATCC 14155 cultures (CRISPRi^−^ aTc^−^) carrying CRISPRi without (CRISPRi^+^ aTc^−^) or with (CRISPRi^+^ aTc^+^) induction of dCas9 protein expression, measured 2 h after addition of 0.2% glucose. aTc, anhydrotetracycline. (C) *hol* promoter activity was measured in an experimental setup by flow cytometry as described for panel A. Results are expressed in arbitrary units (AU). Single dots represent *hol* promoter activities in individual cultures. (D) Growth of E. coli W3110 cells expressing a *pir*-specific CRISPRi system. (E) *hol* promoter activity in E. coli W3110 cells expressing a *pir*-specific CRISPRi system as measured by flow cytometry. Results are expressed in arbitrary units (AU). Means of results from at least three independent replicates are shown; error bars represent standard deviations. *P* values were calculated using the Mann-Whitney test (****, *P < *0.0001; ***, *P < *0.0005; **, *P < *0.005; ns, not significant). Download FIG S4, EPS file, 0.3 MB. Copyright © 2019 Laganenka et al. 2019 Laganenka et al. This content is distributed under the terms of the Creative Commons Attribution 4.0 International license.

10.1128/mBio.01884-19.5 FIG S5 *pir* expression is inhibited by Pir in an AI-2-independent manner in different E. coli phage-free backgrounds. *pir* promoter activity was measured in E. coli BL21(DE3) and ATCC 11303 cells carrying a plasmid-harbored *pir* gene under the control of IPTG-inducible *trc* promoter by flow cytometry. Results are expressed in arbitrary units (AU). Where indicated, 50 μM IPTG was used to induce *pir* expression. Means of results of three independent replicates are shown; error bars represent standard deviations. *P* values were calculated using the Mann-Whitney test (***, *P < *0.0005; **, *P < *0.005; *, *P < *0.05; ns, not significant). Download FIG S5, EPS file, 0.2 MB. Copyright © 2019 Laganenka et al. 2019 Laganenka et al. This content is distributed under the terms of the Creative Commons Attribution 4.0 International license.

We additionally showed that Orf65 is a transcriptional regulator that represses *pir* while positively autoregulating its own expression, whereas the activity of *orf65* promoter is independent of Pir (Fig. S6A to C). No changes in *orf65* expression were detected upon addition of W3110 supernatant or AI-2 (Fig. S6D) or during phage induction (data not shown). Thus, Orf65 is unlikely to play a role in the lysogeny-lysis transition, but *pir* repression by Orf65 might possibly stabilize T1 lysogeny.

10.1128/mBio.01884-19.6 FIG S6 (A) *pir* expression is inhibited by Orf65 in an AI-2-independent manner. *pir* promoter activity was measured in E. coli W3110 carrying plasmid-harbored *orf65* under the control of IPTG-inducible *trc* promoter by flow cytometry. Results are expressed in arbitrary units (AU). (B) *orf65* expression is not affected by Pir. *orf65* promoter activity was measured in E. coli W3110 carrying plasmid-harbored *pir* under the control of IPTG-inducible *trc* promoter by flow cytometry. IPTG (50 μM) was used to induce *pir* expression. Results are expressed in arbitrary units (AU). (C) Orf65 activates its own expression. *orf65* promoter activity was measured in E. coli W3110 carrying plasmid-harbored *orf65* under the control of IPTG-inducible *trc* promoter by flow cytometry. IPTG levels used to induce *pir* are indicated. Results are expressed in arbitrary units (AU). (D) Orf65 activity is not affected by the presence of DPD/AI-2. *orf65* promoter activity was measured in E. coli W3110 carrying plasmid-encoded Orf65 under the control of IPTG-inducible *trc* promoter by flow cytometry. Results are expressed in arbitrary units (AU). Means of results from at least three independent replicates are shown; error bars represent standard deviations. *P* values were calculated using the Mann-Whitney test (***, *P < *0.0005; **, *P < *0.005; *, *P < *0.05; ns, not significant). Download FIG S6, EPS file, 0.3 MB. Copyright © 2019 Laganenka et al. 2019 Laganenka et al. This content is distributed under the terms of the Creative Commons Attribution 4.0 International license.

### PTS-mediated glucose uptake activates *pir* expression via CRP.

**FIG 3:**
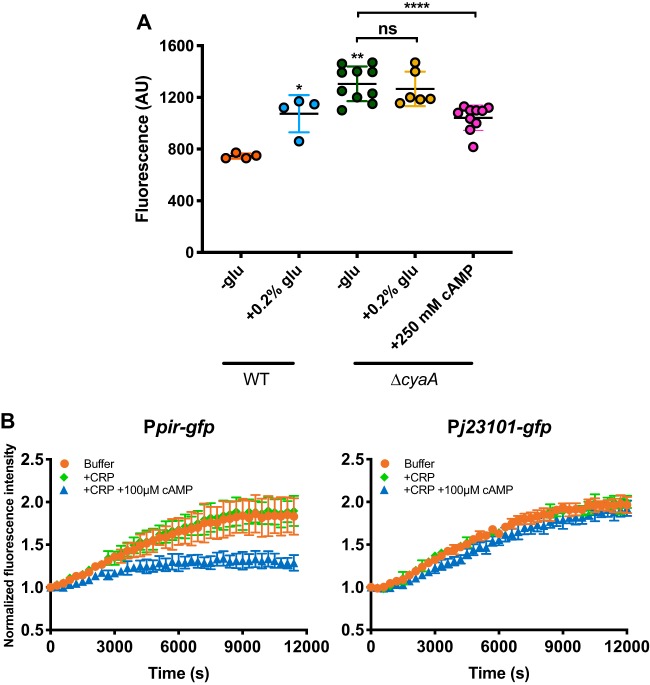
Glucose uptake activates *pir* expression via cAMP-CRP. (A) Deletion of E. coli adenylate cyclase (*cyaA*) results in increased *pir* expression combined with insensitivity to glucose. The effect of *cyaA* deletion can be partially complemented by addition of 250 mM cAMP to the growth medium. Activity of *pir* promoter controlling *gfp* expression was measured by flow cytometry. Results are expressed in arbitrary units (AU). Single dots represent *pir* promoter activities in individual cultures. Means of results from a minimum of four independent replicates are shown; error bars represent standard deviations. *P* value*s* were calculated using the Mann-Whitney test (****, *P < *0.0001; **, *P < *0.005; *, *P < *0.05; ns, not significant). (B) Regulation of *pir* promoter controlling *gfp* by cAMP-CRP in an *in vitro* transcription-translation system (see Materials and Methods for details). GFP fluorescence was measured in a plate reader, and values were normalized to the fluorescence intensity at the time point 0 s. Synthetic constitutive promoter J23101 was used as a negative control. Means of results of three independent replicates are shown; error bars represent standard deviations.

Since elevated *pir* expression was seen upon addition of glucose even in the BL21(DE3) strain lacking the prophage (Fig. 2C), the underlying regulatory mechanism must be provided by the host. Given the dependence of its activity on the PTS signaling, we considered CRP to be a potential regulator. Indeed, abolishing cAMP production in the cell by deleting the adenylate cyclase gene (*cyaA*), which decouples CRP activity from the PTS-mediated sugar uptake, resulted in increased *pir* expression combined with loss of sensitivity to glucose (Fig. 3A). The effect could be partially complemented by supplementing the growth medium with cAMP. These results show that in the absence of glucose, CRP in its cAMP-bound state inhibited *pir* expression and that such inhibition was relieved in the presence of decreasing cAMP levels when glucose was taken up. The repression of the *pir* promoter by cAMP-bound CRP could be directly confirmed using an *in vitro* transcription-translation system (Fig. 3B). Notably, *dam* and *recE* promoters were also inhibited by cAMP-CRP (Fig. S7), suggesting a more global mechanism of CRP-mediated regulation of T1 phage genes.

10.1128/mBio.01884-19.7 FIG S7 cAMP-CRP directly inhibits *dam* and *recE* expression. (A) Activities of *dam* and *recE* promoters in phage-free background strain W3110 were measured by flow cytometry 2 h after addition of 0.2% glucose. Results are expressed in arbitrary units (AU). Means of results of three independent replicates are shown; error bars represent standard deviations. *P* values were calculated using the Mann-Whitney test (*, *P < *0.05). (B) Regulation of *dam* and *recE* promoters controlling *gfp* expression in an *in vitro* transcription-translation system (see Materials and Methods for details). GFP fluorescence was measured in a plate reader, and the values were normalized to the fluorescence intensity seen at time point 0 s. Means of results of three independent replicates are shown; error bars represent standard deviations. Download FIG S7, EPS file, 0.2 MB. Copyright © 2019 Laganenka et al. 2019 Laganenka et al. This content is distributed under the terms of the Creative Commons Attribution 4.0 International license.

## DISCUSSION

Phages are known to employ various distinct strategies to ensure their effective propagation. These can be generally classified into lytic and lysogenic life cycles (13). Virulent (lytic) phages use their hosts for rapid propagation, releasing phage particles into the environment via lysis of the host. In contrast, temperate (lysogenic) phages replicate with their hosts until the extracellular conditions favor phage release and reinfection of the new hosts. Elaborate molecular machineries that control the initial decision between lysis and lysogeny upon host infection, as well as the subsequent lysogeny-lysis decision to enter the lytic cycle, have been described for temperate phages such as lambda (42). However, given the extremely high diversity of bacteriophages in nature, the majority of regulatory mechanisms underlying lysogeny-lysis decisions likely have yet to be discovered.

**FIG 4:**
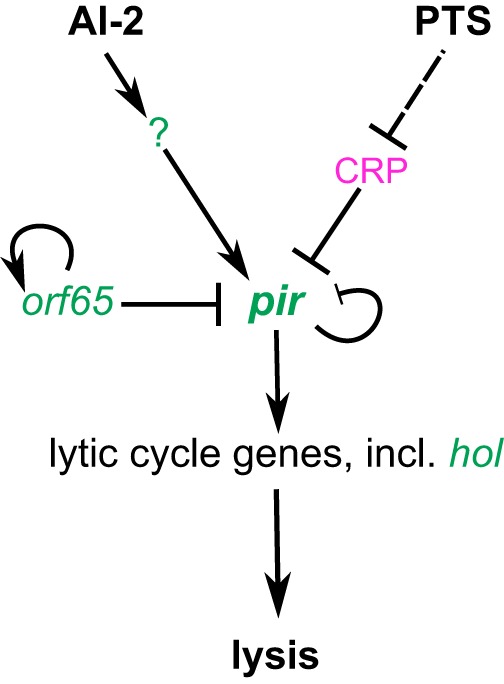
Model of T1 prophage induction in response to AI-2-mediated signaling and sugar influx sensing. Phage-encoded Pir is a transcription regulator controlling expression of genes required for T1 prophage induction. In the absence of external stimuli, low *pir* expression is ensured by the self-inhibitory activity of Pir along with inhibition by Orf65 and by cAMP-CRP. Activation by glucose, likely mediated by PTS, relieves CRP inhibition of *pir* expression. This in turn results in Pir-dependent activation of lytic cycle and accumulation of holin (*hol*), followed by cell lysis and phage release. Expression of *pir* is also upregulated in response to AI-2 by an as-yet-unknown mechanism, similarly initiating prophage induction. Phage-carried genes are marked in green; host-encoded CRP is marked in pink. Regulatory effects are shown by solid lines (transcriptional regulation) or dashed lines (posttranslational regulation). Positive and negative regulatory effects are indicated by lines with arrowheads and by lines with blunt ends, respectively.

Here, we describe a novel lysogenic state of T1 phage, which has been previously viewed as a typical virulent phage, showing that this phage can be stably propagated in the extrachromosomal state in E. coli host cells. Furthermore, our results suggest that this prophage state integrates information about bacterial population density and the metabolic state of the host to control its switch from lysogeny to lysis. This regulation apparently occurs at the level of a phage-encoded transcriptional regulator, Pir (Fig. 4). In the absence of external stimuli, a steady state of *pir* expression is ensured by the self-inhibitory regulation mediated by Pir, along with additional inhibition of its promoter by Orf65 and by cAMP-CRP. Increased sugar influx relieves CRP-mediated inhibition of *pir* expression, resulting in Pir-dependent activation of the lytic cycle. *pir* is similarly upregulated in the presence of AI-2, likely explaining the dependence of lysis on bacterial culture density. Although the precise mechanism of AI-2-mediated prophage induction remains unknown, our observation that transcription of *pir* is induced by AI-2 in prophage-carrying strain ATCC 15144 but not in prophage-free strain BL21(DE3) suggests that the regulatory factor(s) involved—possibly in the form of a small RNA or a riboswitch—are encoded by the phage genome.

Since the concentration of quorum-sensing molecules is essentially a function of bacterial population density (43), it would be expected that lysogenic phages have evolved mechanisms to adopt this signal for their lysogeny-lysis decisions. Indeed, recent studies have shown that quorum sensing led to prophage induction in Vibrio cholerae and Enterococcus faecalis (24, 25). In the case of V. cholerae, induction occurred in response to the presence of 3,5-dimethylpyrazin (DPO), an intraspecies signaling molecule, and was mediated by a homologue of the host quorum-sensing activator VqmA (24). In the case of E. faecalis, prophage induction mediated by AI-2 was observed, similarly to the case described here for E. coli, but the underlying regulatory circuit was not investigated (25).

Equally interesting is the ability of T1 phage to effectively respond to changes in host metabolism. Although the nutrition state is known to affect lysis-lysogeny decisions (15, 42, 44–46), this is the first example of the phage being able to “eavesdrop” on the host’s metabolic signaling system to decide whether the conditions are favorable for prophage induction. Given that Pir homologues are found in phages infecting not only E. coli but also *Salmonella*, *Citrobacter*, *Klebsiella*, *Cronobacter*, and *Pantoea* species (see Table S3 in the supplemental material), the mechanisms of prophage induction described in this report may be common among *Enterobacteriaceae* viruses.

## MATERIALS AND METHODS

### Bacterial strains and culture conditions.

All strains and plasmids used in this study are listed in Table 1. E. coli strains were grown in liquid tryptone broth (TB) medium (10 g tryptone and 5 g NaCl per liter) or in lysogeny broth (LB) medium (10 g tryptone, 10 g NaCl, and 5 g yeast extract per liter) supplemented with antibiotics where necessary.

**TABLE 1:** List of bacterial strains and plasmids used in this study^*a*^

Strain or plasmid	Relevant genotype or phenotype	Source and/or reference
Strains		
E. coli ATCC 15144	Wild-type strain	Leibniz Institute DSMZ—German Collection of Microorganisms and Cell Cultures, Braunschweig, Germany
E. coli ECOR1-72	The E. coli reference collection of natural isolates	STEC Center, Michigan State University, USA
E. coli BL21(DE3)	F^−^ *ompT gal dcm lon hsdS*_B_(r_B_^–^ m_B_^–^) λ(DE3 [*lacI lacUV5*-*T7p07* *ind1 sam7 nin5*]) [*malB*^+^]_K-12_(λ^S^)	Laboratory collection
E. coli ATCC 11303	Wild-type strain for T1 phage propagation	Leibniz Institute DSMZ—German Collection of Microorganisms and Cell Cultures (Braunschweig, Germany)
E. coli MG1655	F^−^ lambda *ilvG rfb*-50 *rph*-*1*	Laboratory collection
E. coli YC55	MG1655 Δ*cyaA* Km^s^	This work
E. coli YC170	MG1655 Δ*cyaA* Δ*crp* Km^r^	This work
E. coli W3110	W3110 derivative with functional RpoS [*rpoS396*(*Am*)]	54
E. coli VS823	W3110 Δ*luxS* Km^s^	29

Plasmids		
pTrc99A	Amp^r^; expression vector; pBR ori; *trc* promoter, IPTG inducible	55
pUA66	Km^r^; expression vector; SC101 ori, GFPmut2 under the control of promoter of interest	56
pLeoL9	Amp^r^; *cor* in pTrc99A, IPTG inducible	This work
pLeoL10	Amp^r^; *pir* in pTrc99A, IPTG inducible	This work
pLeoL11	Amp^r^; *orf65* in pTrc99A, IPTG inducible	This work
pLeoL12	Km^r^; P*hol-gfp* in pUA66	This work
pLeoL13	Km^r^; P*dam-gfp* in pUA66	This work
pLeoL14	Km^r^; P*pir-gfp* in pUA66	This work
pLeoL15	Km^r^; P*recE-gfp* in pUA66	This work
pLeoL16	Km^r^; P*orf65-gfp* in pUA66	This work
pVS1723	Km^r^; P*lsr-gfp* in pUA66	29
pYC75	Cam^r^; *crp* with C-terminal His6 tag under the control of IPTG- inducible T5-*lac* promoter in pCA24N expression vector	This work and reference 57
pAL60	Cam^r^, *gfp* under the control of a synthetic constitutive promoter J23101 in pSB1C3 expression vector	This work and Anderson Promoter Collection
pdCas9	aTc-inducible expression of a catalytically inactive bacterial Cas9 (Streptococcus pyogenes) for bacterial gene knockdown	Addgene, USA; catalogue no. 44249
pgRNA	Expression of customizable guide RNA (gRNA) for bacterial gene knockdown	Addgene, USA; catalogue no. 44251

a pgRNA, pregenomic RNA; Km^s^, kanamycin sensitive; Km^r^, kanamycin resistant; Amp^r^, ampicillin resistant; Cam^r^, chloramphenicol resistant.

### Plasmids.

Genes of T1 phage were amplified from the purified T1 phage DNA using primers listed in Table S1 in the supplemental material. The PCR products were purified, digested with SacI and XbaI enzymes (NEB, USA), and ligated into pTrc99A expression vector. For construction of fluorescent reporters, promoter regions (up to 150 bp upstream and 15 bp downstream of the start codon) of genes of interest were amplified from the purified T1 phage DNA and cloned into pUA66 vector using XhoI and BamHI cloning sites.

10.1128/mBio.01884-19.8 TABLE S1 List of oligonucleotides used in this study. Download Table S1, DOCX file, 0.1 MB. Copyright © 2019 Laganenka et al. 2019 Laganenka et al. This content is distributed under the terms of the Creative Commons Attribution 4.0 International license.

10.1128/mBio.01884-19.9 TABLE S2 List of ORFs present in E. coli ATCC 15144 but absent in E. coli BL21(DE3). Download Table S2, DOCX file, 0.07 MB. Copyright © 2019 Laganenka et al. 2019 Laganenka et al. This content is distributed under the terms of the Creative Commons Attribution 4.0 International license.

10.1128/mBio.01884-19.10 TABLE S3 List of Pir homologues identified by a BLAST search using the blastp algorithm. Download Table S3, DOCX file, 0.1 MB. Copyright © 2019 Laganenka et al. 2019 Laganenka et al. This content is distributed under the terms of the Creative Commons Attribution 4.0 International license.

### CRISPR interference.

CRISPR interference-mediated inhibition of *pir* (*orf23*) expression was performed as described previously (40) using pdCas9 (Addgene, USA; catalogue number 44249) and sgRNA (Addgene, USA; catalogue number 44251) plasmids (41). The guide RNA (gRNA) plasmid was customized by site-directed mutagenesis by the use of forward primer EcF_LS2, carrying the 20-nucleotide (nt) base-pairing sequence (5′-TAGAACCCGCAACGCTGGCG-3′), and reverse primer EcR (see Table S1). The base pairing region was designed to target the *pir* gene on T1 phage DNA on the coding strand adjacent to protospacer motif AGG. dCas9 protein expression was induced with 5 ng/ml aTc in both overnight and day cultures.

### Real-time quantitative PCR (RT-qPCR).

Total mRNA isolation and RT-qPCR for confirmation of CRISPRi-mediated *pir* mRNA depletion were carried out as described previously (47). A 200-ng volume of total mRNA was used in every reaction. Primers used for *pir* and *cor* mRNA detection in the samples are listed in Table S1.

### *In vitro* transcription-translation assay.

Regulation of *pir*, *dam*, and *recE* promoters controlling *gfp* expression mediated by CRP was assayed as described previously (48, 49). Cell-free lysates were prepared by sonication from the E. coli MG1655 Δ*cyaA* Δ*crp* strain, in order to avoid contamination with cellular cAMP and CRP. CRP was purified from E. coli strain BL21(DE3) carrying plasmid pYC75 expressing CRP with a C-His6 tag under the control of a T5-*lac* promoter using Protino Ni-TED packed columns (Machery Nagel, Germany; catalogue number 745100.50). Purified protein was concentrated by filtration with an Amicon Ultra-0.5 centrifugal filter unit (Millipore, Germany; catalogue number UFC501008). The promoter assay reaction mixture (10 μl) contained reaction buffer (10 mM magnesium glutamate; 10 mM ammonium glutamate; 130 mM potassium glutamate; 1.2 mM ATP; 0.850 mM [each] GTP, UTP, and CTP; 0.034 mg/ml folinic acid; 0.171 mg/ml yeast tRNA; 2 mM amino acids; 30 mM PEP; 0.33 mM NAD; 0.27 mM coenzyme A [CoA]; 4 mM oxalic acid; 1 mM putrescine; 1.5 mM spermidine; 57 mM HEPES), 30% cell-free lysate, 5 ng/μl of plasmids containing promoter fusions of interest, and 0.5 μl RNase inhibitor (Invitrogen, USA; catalogue number AM2694). The cAMP and CRP concentrations were 100 μM and 100 μg/ml, respectively. GFP fluorescence was measured at 10-min intervals with an Infinite M Nano+ plate reader (Tecan Group, Switzerland) at 37°C. As a negative control, plasmid pAL60 carrying synthetic constitutive promoter J23101 (Anderson Promoter Collection, http://parts.igem.org/Promoters/Catalog/Anderson) fused to *gfp* was used.

### T1 prophage induction experiments and phage titer determination.

E. coli strain ATCC 15144 carrying T1 prophage was grown overnight in 5 ml TB at 37°C with shaking. Day cultures were prepared by diluting the overnight cultures 100 times in 2 ml TB and were grown at 37°C with shaking (220 rpm) for 1 h. Synthetic DPD/AI-2 (27) and glucose or 2-deoxy-d-glucose (Sigma-Aldrich, Germany) were added for final concentrations of 30 μM and 0.2%, respectively, followed by further incubation at 37°C with shaking. As an alternative to DPD/AI-2, 10 μl of E. coli W3110 cell-free supernatant was used. Prophage induction and the resulting cell lysis were visually observed after 60 to 130 min by the dramatic drop in the optical density (OD) of the test culture (OD at 600 nm [OD_600_], approximately 0.1 to 0.2) compared to that of the control (OD_600_, approximately 0.6 to 0.9). The phage titer after prophage induction and E. coli culture lysis was determined as the PFU level per milliliter using the Gratia method (50).

### Genomic DNA extraction, sequencing, and analysis.

E. coli ATCC 15144 was grown overnight in 5 ml LB at 37°C with shaking. The cells were spun down (5 min, 4,000 rpm), and the genomic DNA was extracted using a NucleoSpin microbial DNA purification kit (Macherey-Nagel, Germany; catalogue number 740235.50). T1 phage DNA was extracted upon DPD/AI-2-induced prophage induction and cell lysis using a phage DNA isolation kit (Norgen Biotek Corp., Canada; catalogue number 46800).

DNA concentration was measured using a Qubit 4 fluorometer (Invitrogen, USA; catalogue number Q3326) and a Qubit double-stranded DNA (dsDNA) high-sensitivity (HS) assay kit (Invitrogen, USA; catalogue number Q322851). The DNA library for sequencing was prepared using a NEBNext Ultra II FS DNA library preparation kit for Illumina (NEB, USA; catalogue number 3E7805L) and NEBNext multiplex oligonucleotides for Illumina (96 Index Primers) (NEB, USA; catalogue number E6609S). Library quality was verified using an Agilent Technology 2100 Bioanalyzer (Agilent Technologies, USA) and an Agilent high-sensitivity DNA kit (Agilent Technologies, USA; catalogue number 5067-4626). DNA was sequenced with an Illumina MiniSeq system (Illumina, USA).

Quality controlled, trimmed reads were assembled *de novo* into sequences of contigs with >100-fold coverage (for the E. coli DSM 15144 genome) and into a single contig with >4,000-fold coverage (for T1 phage genome) using Geneious 12.0 (*N*_50_ = 114904) (51). The absence of T1 phage sequences in the bacterial genome was confirmed using nucleotide BLAST against the GenBank database and additionally by analyzing the sequence with PHASTER (Phage Search Tool Enhanced Release) (52). The complete T1 phage genome was analyzed for the presence of bacterial sequences flanking its terminal ends.

### Transmission electron microscopy.

Transmission electron microscopy (performed with a JEM-1400 transmission electron microscope [Jeol, USA]) was used to visualize phage particles in E. coli ATCC 15144 lysates as described previously (53).

### Flow cytometry.

Promoter activities of *orf13* (*hol*), *orf20* (*dam*), *orf23* (*pir*), *orf29* (*recE*), and *orf65* were assayed using plasmid-based reporters containing the respective promoter regions fused to *gfp* (see the description of the molecular cloning techniques for more details). Bacterial cultures were grown in TB as described above (supplemented with IPTG or 5 ng/ml aTc where necessary) to an OD_600_ of 0.6 or until the onset of cellular lysis during the prophage induction. The samples were then diluted 1:100 in tethering buffer (10 mM KH_2_PO_4_, 100 μM EDTA, 1 μM l-methionine, 10 mM sodium lactate, pH 7.0), and fluorescence was measured with a BD LSRFortessa SORP cell analyzer (BD Biosciences, Germany). AI-2 quantification in culture supernatants was performed as described previously using a biosensor strain deficient in AI-2 production (29).

### Data availability.

The contigs used in this work were deposited in GenBank under accession number PRJNA526015. T1 phage genome sequence data determined in this work were deposited in GenBank under accession number MN153797.
