# Supplementary material for: Quorum Sensing and Metabolic State of the Host Control Lysogeny-Lysis Switch of Bacteriophage T1
Source: mBio. 2019 Sep 10;10(5):e01884-19. doi: 10.1128/mBio.01884-19 (PMC6737242; doi:10.1128/mBio.01884-19)
Supplement: TABLE S1 [file mBio.01884-19-st001.docx]

| **Primer name** | **Primer sequence (5’→3’)** | **Purpose** |
| --- | --- | --- |
| dcor_FWD | ATGAAAAAGTTAATCACCGTTATCGC | Diagnostic primer for *cor* gene detection |
| dcor_REV | TTATATCGAGCAATCGTGAGATTTGAAG |  |
| dorf47_FWD | GTATCTGATCCAGGCTGGCGTAA | Diagnostic primer for *orf47* gene detection |
| dorf47_REV | TTACACGCCAGTGATTAAGACAATAGT |  |
| cor_FWD | **CGGAGCTCG**ATGAAAAAGTTAATCACCGTTATCGC  SacI | Cloning of *cor* into pTrc99A vector |
| cor_REV | **GCTCTAGAGC**TTATATCGAGCAATCGTGAGATTTGAAG  XbaI |  |
| pir_FWD | **CGGAGCTCG**ATGAGTATTCAACGTATCGCAGAG  SacI | Cloning of *pir* into pTrc99A vector |
| pir_REV | **GCTCTAGAG**CTTAACTAAAAATGCTATTGGTCGTTCC  XbaI |  |
| orf65_FWD | **CGGAGCTCG**ATGAAACACTTAATCTGCATTGAAGC  SacI | Cloning of *orf65* into pTrc99A vector |
| orf65_REV | **GCTCTAGAGC**TTACCAGCAACTGATCTCATAGTTGGC  XbaI |  |
| Phol_FWD | **CCGCTCGAGCGG**ATTTGAGATTTACGACGGGCGA  XhoI | Cloning of *hol* promoter region into pUA66 vector |
| Phol_REV | **CGCGGATCCGCG**CAGCCGTTAAAAACTCTTTCATAATCT  BamHI |  |
| Pdam_FWD | **CCGCTCGAGCGG**CAAAGGAAAGGCGTCACCAGTCAG  XhoI | Cloning of *dam* promoter region into pUA66 vector |
| Pdam_REV | **CGCGGATCCGCG**ATCATTAAAGTCTTTCATTTTTATTCCTCTCG  BamHI |  |
| Ppir_FWD | **CCGCTCGAGCGG**TCCTGAGTTCTCTTTATATAGTCATCGCC  XhoI | Cloning of *pir* promoter region into pUA66 vector |
| Ppir_REV | **CGCGGATCCGCG**TCTGCGATACGTTGAATACTCATCTT  BamHI |  |
| PrecE_FWD | **CCGCTCGAGCGG**CTGTATTTCTAATAAAAAGCGGTTACAAGT  XhoI | Cloning of *recE* promoter region into pUA66 vector |
| PrecE_REV | **CGCGGATCCGCG**GTGAATACCTGGAACATTGCGTTTATCT  BamHI |  |
| Porf65_FWD | **CCGCTCGAGCGG**TTAAAACGAATAGCACGAATTGC  XhoI | Cloning of *orf65* promoter region into pUA66 vector |
| Porf65_REV | **CGCGGATCCGCG**GCAGATTAAGTGTTTCATGATCGCTT  BamHI |  |
| EcF_LS2 | TAGAACCCGCAACGCTGGCGGTTTTAGAGCTAGAAATAGCA  AGTTAAAATAAGGC | Cloning of pgRNA for CRISPRi |
| EcR | ACTAGTATTATACCTAGGACTGAGCTAGC |  |
| RTpir_FWD | CAGAGTCAACTGGTGAAATCGATAAA | RT-qPCR, *pir* mRNA quantification |
| RTpir_REV | CGTAATATTCGATAAGGGCATCAAGTC |  |
| RTcor_FWD | TTATCGCTGCGGCATTCATCC | RT-qPCR, *cor* mRNA quantification |
