# Supplementary material for: Quorum Sensing and Metabolic State of the Host Control Lysogeny-Lysis Switch of Bacteriophage T1
Source: mBio. 2019 Sep 10;10(5):e01884-19. doi: 10.1128/mBio.01884-19 (PMC6737242; doi:10.1128/mBio.01884-19)
Supplement: TABLE S2 [file mBio.01884-19-st002.docx]

| **ORF product** |
| --- |
| IS3 family transposase [*Shigella flexneri*] |
| hypothetical protein ECB_01341 [*Escherichia coli* B str. REL606] |
| bacteriophage N4 receptor [*Escherichia coli* B185] |
| bacterial group 1 Ig-like protein [*Escherichia coli* MS 187-1] |
| intimin-like adhesin FdeC, partial [*Escherichia coli*] |
| Membrane-bound lytic murein transglycosylase B, partial [*Escherichia coli* DORA_A_5_14_21] |
| aconitase family protein [*Escherichia coli HS*] |
| type VI secretion system tip protein VgrG [*Escherichia coli*] |
| phage tail protein, partial [*Escherichia coli*] |
| putative antigen 43 precursor (fluffing protein) |
| type II secretion system protein GspD [*Escherichia coli*] |
| phage tail protein [*Escherichia coli*] |
| putative prophage protein [*Escherichia coli*] |
| replication endonuclease [*Escherichia coli*] |
| type IV secretion protein Rhs [*Escherichia coli*] |
| exodeoxyribonuclease VIII [*Escherichia coli*] |
| host specificity protein J [*Escherichia coli*] |
| exonuclease [*Escherichia coli*] |
| FeS assembly protein SufB [*Escherichia coli* D9] |
| type IV secretion protein Rhs, partial [*Escherichia coli*] |
