# Supplementary material for: Quorum Sensing and Metabolic State of the Host Control Lysogeny-Lysis Switch of Bacteriophage T1
Source: mBio. 2019 Sep 10;10(5):e01884-19. doi: 10.1128/mBio.01884-19 (PMC6737242; doi:10.1128/mBio.01884-19)
Supplement: TABLE S3 [file mBio.01884-19-st003.docx]

| **Accession number** | **Description** | **Score (Bits)** | **E value** | **Max. identity** |
| --- | --- | --- | --- | --- |
| YP_003922 | hypothetical protein T1p23 [*Escherichia* virus T1] | 311 | 4e-107 | 100% |
| YP_009112952 | hypothetical protein pSf2_014 [*Shigella* phage pSf-2] | 310 | 1e-106 | 99% |
| YP_004414874 | hypothetical protein Shfl1p58 [*Shigella* virus Shfl1] | 307 | 1e-105 | 98% |
| ALT58258 | hypothetical protein JMPW1_054 [*Escherichia* phage JMPW1] | 306 | 2e-105 | 98% |
| AWD90909 | hypothetical protein [*Enterobacteria* phage vB_EcoS_IME18] | 306 | 2e-105 | 97% |
| AWY08657 | hypothetical protein [*Escherichia* phage Eco_BIFF] | 306 | 3e-105 | 97% |
| YP_007112731 | hypothetical protein B508_00330 [*Escherichia* phage ADB-2] | 306 | 4e-105 | 97% |
| ATN48462 | hypothetical protein Sf11_59 [*Shigella* phage Sfin-1] | 304 | 2e-104 | 97% |
| ATN94091 | hypothetical protein FLXISF001_013 [*Shigella* phage vB_SflS-ISF001] | 272 | 9e-92 | 89% |
| AWD92261 | putative transcriptional regulator [*Enterobacteria* phage vB_EcoS_IME347] | 252 | 6e-84 | 79% |
| QAX92072 | hypothetical protein vBSsoS008_065 [*Shigella* phage vB_SsoS_008] | 244 | 5e-81 | 94% |
| ASV44806 | hypothetical protein vBEcoS95_02 [*Escherichia* phage vB_EcoS-95] | 165 | 1e-49 | 58% |
| SMH63968 | putative transcriptional regulator [*Escherichia* phage vB_Eco_swan01] | 165 | 2e-49 | 58% |
| YP_001285547 | gp57 [Escherichia virus TLS] | 160 | 6e-47 | 58% |
| AWY04361 | putative transcriptional regulator [*Escherichia* phage LL5] | 160 | 6e-47 | 58% |
| AGF87911 | hypothetical protein SP126_00405 [*Salmonella* virus SP126] | 160 | 8e-47 | 58% |
| ARB06704 | putative transcriptional regulator [*Salmonella* phage GJL01] | 159 | 1e-46 | 58% |
| AVQ09770 | putative transcriptional regulator [*Salmonella* phage vB_SenS_PHB07] | 159 | 1e-46 | 58% |
| AXY85484 | hypothetical protein CPT_Sazh_060 [*Citrobacter* phage Sazh] | 159 | 1e-46 | 58% |
| YP_009280763 | hypothetical protein [*Salmonella* phage phSE-2] | 159 | 1e-46 | 58% |
| YP_009148735 | transcriptional regulator [*Citrobacter* virus Stevie] | 157 | 1e-45 | 59% |
| YP_009284683 | putative transcriptional regulator [*Enterobacteria* phage vB_EcoS_NBD2] | 151 | 6e-44 | 56% |
| YP_008059724 | hypothetical protein pSf1_002 [*Shigella* phage pSf-1] | 149 | 3e-43 | 60% |
| YP_009284866 | putative transcriptional regulator [*Klebsiella* phage PKP126] | 147 | 1e-42 | 54% |
| YP_006987868 | putative transcriptional regulator [*Escherichia* phage vB_Eco_ACG-M12] | 146 | 9e-42 | 51% |
| AOZ65315 | putative transcriptional regulator [*Klebsiella* phage vB_KpnS_KpV522] | 144 | 1e-41 | 53% |
| AYJ73357 | transcriptional regulator [*Cronobacter* phage CS01] | 142 | 2e-40 | 52% |
| YP_007112250 | putative transcriptional regulator [*Enterobacteria* phage vB_EcoS_Rogue1] | 141 | 3e-40 | 54% |
| YP_009036037 | putative transcriptional regulator [*Escherichia* phage e4/1c] | 141 | 4e-40 | 54% |
| YP_007005416 | transcriptional regulator [*Cronobacter* virus Esp2949-1] | 140 | 6e-40 | 51% |
| YP_277513 | hypothetical transcriptional regulator [*Escherichia* virus KP26] | 140 | 7e-40 | 53% |
| YP_009018636 | gp22 [Escherichia phage EB49] | 140 | 7e-40 | 53% |
| ATE85751 | lambda repressor-like DNA binding protein [*Shigella* phage Sf12] | 140 | 2e-39 | 53% |
| AQY55307 | hypothetical protein ESCO41_00080 [*Escherichia* phage vB_EcoS_ESCO41] | 136 | 6e-38 | 53% |
| YP_398996 | putative transcriptional regulator [*Escherichia* virus Rtp] | 135 | 8e-38 | 52% |
| QAU04375 | putative transcriptional regulator [*Enterobacteria* phage vB_EcoS_IME542] | 135 | 1e-37 | 52% |
| AQN32376 | transcriptional regulator [*Escherichia* phage vB_Ecos_CEB_EC3a] | 135 | 2e-37 | 52% |
| ATE86081 | transcriptional regulator [*Shigella* phage Sd1] | 134 | 3e-37 | 51% |
| ATW61832 | hypothetical protein [*Escherichia* phage DTL] | 134 | 3e-37 | 51% |
| AZS06344 | putative transcriptional regulator [*Pantoea* phage vB_PagS_AAS23] | 133 | 4e-37 | 51% |
| AXQ68029 | putative transcriptional regulator [*Klebsiella* phage NJS2] | 129 | 2e-35 | 55% |
| YP_009197873 | putative transcriptional regulator [*Klebsiella* phage 1513] | 127 | 6e-35 | 54% |
| YP_009195388 | putative transcriptional regulator [*Klebsiella* phage KLPN1] | 127 | 1e-34 | 53% |
| YP_009226016 | putative transcriptional regulator [*Klebsiella* phage KP36] | 127 | 1e-34 | 54% |
| ATN93778 | hypothetical protein [*Escherichia* phage SRT8] | 124 | 2e-34 | 75% |
| AEQ39200 | hypothetical protein [*Enterobacter* virus F20] | 124 | 2e-33 | 53% |
| YP_009223488 | integrase [*Salmonella* phage 36] | 87 | 7e-20 | 59% |
| WP_082818992 | hypothetical protein [*Cupriavidus nantongensis*] | 40 | 0.22 | 56% |
| WP_118073856 | hypothetical protein [*Novosphingobium* *sp*. THN1] | 38 | 0.55 | 40% |
| WP_115937728 | hypothetical protein [*Aestuariispira insulae*] | 40 | 0.82 | 33% |
| APC46561 | transcriptional regulator [*Alteromonas* phage PB15] | 38 | 0.86 | 51% |
| WP_042111230 | hypothetical protein [*Pseudomonas putida*] | 36 | 4 | 41% |
| ACA71814 | hypothetical protein PputW619_1309 [*Pseudomonas putida* W619] | 36 | 4.6 | 41% |
| WP_035961149 | ATP-binding protein [*Kocuria marina*] | 37 | 6.9 | 46% |
| WP_088870725 | hypothetical protein [*Nitrospirillum amazonense*] | 37 | 8.8 | 41% |
| ASG19759 | hypothetical protein Y958_02140 [*Nitrospirillum amazonense* CBAmc] | 37 | 8.9 | 41% |
